# Mitophagy Protects Against Cisplatin-Induced Injury in Granulosa Cells

**DOI:** 10.3390/toxics13050332

**Published:** 2025-04-23

**Authors:** Sihui Zhu, Mingge Tang, Jiahua Chen, Shuhang Li, Rufeng Xue

**Affiliations:** 1 Reproductive Medicine Center, Department of Obstetrics and Gynecology, The First Affiliated Hospital of Anhui Medical University, Hefei 230088, China; zhusihui124@163.com; 2 NHC Key Laboratory of Study on Abnormal Gametes and Reproductive Tract, Anhui Medical University, Hefei 230032, China; 3 Key Laboratory of Population Health Across Life Cycle, Anhui Medical University, Ministry of Education of the People’s Republic of China, Hefei 230032, China; 4 Anhui Province Key Laboratory of Reproductive Disorders and Obstetrics and Gynaecology Diseases, Hefei 230032, China; tmgdingding@outlook.com (M.T.);; 5 Biopreservation and Artificial Organs, Anhui Provincial Engineering Research Center, Anhui Medical University, Hefei 230032, China; 6 Institute of Health and Medicine, Hefei Comprehensive National Science Center, Hefei 230031, China

**Keywords:** cisplatin, mitophagy, apoptosis, KGN cells, melatonin

## Abstract

Cisplatin, a widely used chemotherapeutic agent, is known to induce premature ovarian insufficiency (POI) and infertility in women of reproductive age. Among the contributing factors, cisplatin-induced apoptosis of ovarian granulosa cells is considered a primary driver of ovarian dysfunction; however, the underlying mechanisms remain incompletely understood. In this study, we investigated the cytotoxicity of cisplatin on the granulosa cell line KGN in vitro and explored the associated mechanisms. Our results demonstrate that cisplatin induces KGN cell apoptosis in a dose-dependent manner and impairs mitochondrial function, as evidenced by excessive ROS production, membrane potential collapse, and reduced ATP synthesis. Mitophagy, a key cellular self-protection mechanism that selectively removes damaged mitochondria, was activated following cisplatin treatment, mitigating its detrimental effects on KGN cells. Activation of mitophagy with urolithin A (UA) ameliorated cisplatin-induced mitochondrial dysfunction and apoptosis, whereas inhibition of mitophagy with cyclosporine A (CsA) exacerbated these effects. Furthermore, pretreatment with the clinical drug melatonin significantly enhanced mitophagy, effectively attenuating cisplatin-induced apoptosis in KGN cells. This study proposes a novel therapeutic strategy for patients undergoing tumor chemotherapy, aiming to preserve treatment efficacy while reducing the adverse effects of chemotherapeutic agents on ovarian function, thereby improving patients’ quality of life.

## 1. Introduction

Cisplatin is a widely used chemotherapeutic agent known for its efficacy in treating a broad spectrum of malignant tumors [1]. However, the application of it in clinical settings is frequently constrained by the severe adverse effects it exerts on healthy tissues, including neurotoxicity, nephrotoxicity, and ototoxicity [2,3]. Among these adverse effects, cisplatin-induced damage to reproductive tissues, particularly the ovaries, has garnered increasing attention due to its impact on female fertility.

The mammalian ovary is a critical reproductive organ responsible for producing competent oocytes, which are essential for female fertility [4]. With the rising incidence of gynecological malignancies and the trend toward delayed childbearing, chemotherapy-induced premature ovarian failure (POF) has become a significant clinical concern [5]. POF is characterized by a depleted ovarian reserve and a reduced number of ovarian follicles, which are closely linked to granulosa cell dysfunction and apoptosis [6,7,8]. Granulosa cells are indispensable in several critical aspects of ovarian biology. They play a fundamental role in the development and maturation of oocytes, as well as in the survival of follicles. Consequently, the maintenance of their structural integrity is essential for the preservation of overall ovarian function [9]. Recent studies have demonstrated that cisplatin suppresses granulosa cell proliferation and induces apoptosis through mechanisms involving oxidative stress, mitochondrial dysfunction, and cell cycle arrest [10,11]. In detail, cisplatin increases the level of pro-apoptotic Bax expression and concurrently decreases the level of anti-apoptotic Bcl-2 expression. This shift in expression patterns results in reduced viability of granulosa cells, ultimately leading to a decrease in ovarian reserve [12]. Despite these advances, the precise molecular pathways underlying cisplatin-induced granulosa cell damage and ovarian toxicity remain incompletely understood. Further studies are necessary to unravel these mechanisms and determine potential therapeutic interventions that can reduce cisplatin-induced ovarian injury while ensuring its antitumor effectiveness remains intact.

Mitochondria, the central hubs of cellular energy metabolism and signaling, are critical targets for platinum-based chemotherapeutic agents, which induce apoptosis by disrupting mitochondrial function [13]. These agents often lead to a decline in mitochondrial membrane potential and an accumulation of reactive oxygen species (ROS), triggering mitophagy—a selective autophagic process that removes damaged or dysfunctional mitochondria [14]. Mitophagy is crucial for sustaining both the quality and the number of mitochondria, with its malfunction being tightly linked to the development of chronic illnesses, including neurodegenerative and cardiovascular disorders [15,16]. Although moderate mitophagy supports mitochondrial integrity, the precise mechanisms governing the selective targeting of impaired mitochondria remain incompletely understood. Recent research has emphasized the central significance of the PINK1–Parkin system. In this pathway, PINK1 accumulates specifically on mitochondria that are damaged, prompting the recruitment and subsequent activation of Parkin. This, in turn, triggers ubiquitination events, which serve to label mitochondria for degradation via autophagy [17,18]. Notably, PINK1–Parkin-mediated mitophagy has been shown to mitigate cisplatin-induced nephrotoxicity [19]. However, the role of mitophagy in cisplatin-induced reproductive toxicity, particularly in ovarian granulosa cells, remains poorly explored.

Produced by the pineal gland, melatonin, an endogenous hormone, has attracted attention due to its wide array of biological activities. These include its antioxidant capabilities, anti-inflammatory effects, and antitumor properties [20]. Recent evidence suggests that melatonin can modulate mitophagy, offering cytoprotective effects in various pathological contexts [21]. For instance, melatonin has been shown to regulate mitophagy in polycystic ovary syndrome (PCOS) by mitigating oxidative stress and restoring mitochondrial function [22]. Additionally, melatonin enhances mitochondrial performance in renal injury by promoting PINK1- and Parkin-dependent mitophagy [23]. Despite these recent developments, the extent to which melatonin safeguards against cisplatin-induced reproductive harm, particularly in the context of ovarian granulosa cells, has not undergone exhaustive examination.

In this study, we explored the role of mitophagy in cisplatin-induced apoptosis in KGN cells, a model of human granulosa cells. By establishing a cisplatin-induced apoptosis model, we examined changes in mitophagy and evaluated its protective effects using mitophagy inhibitors and inducers. Furthermore, we investigated the potential of melatonin as a mitophagy activator to mitigate cisplatin-induced reproductive toxicity. Our findings provide new insights into the molecular mechanisms underlying cisplatin’s side effects and offer a novel therapeutic strategy to enhance chemotherapy regimens by reducing the adverse impacts of chemotherapeutic agents on reproductive tissues.

## 2. Materials and Methods

### 2.1. Reagents and Antibodies

Cisplatin (HY-17394), Urolithin A (HY-100599), Cyclosporin A (HY-B0579), DMSO (HY-Y0320), and DMF (HY-Y0345) were purchased from MedChemExpress (Monmouth Junction, NJ, USA). Melatonin (CAS 73-31-4, purity ≥ 98%) was purchased from Sigma Aldrich (St. Louis, MO, USA). The primary antibodies included Anti-Bax (1:1000, D2E11, CST, Danvers, MA, USA), Anti-Bcl2 (1:1000, 15071S, CST, Danvers, MA, USA), Cleaved caspase-3 (1:500, 9664L, CST, Danvers, MA, USA), Anti-beta-Actin (1:1000, 3700S, CST, Danvers, MA, USA), Anti-PINK1 (1:2000, 23274-1-AP, Proteintech, Wuhan, China), Anti-Parkin (1:1000, 14060-1-AP, ZEN-BIOSCIENCE, Chengdu, China), Anti-LC3II/LC3I (1:1000, 12741S, CST, Danvers, MA, USA), Anti-P62 (1:1000, 29503-1-AP, Proteintech, Wuhan, China), and Anti-GAPDH (1:1000, 10494-1-AP, Proteintech, Wuhan, China). Secondary antibodies, including anti-rabbit (1:10,000, SA00001-2) and anti-mouse (1:10,000, SA00001-1), were obtained from Proteintech (Wuhan, China).

### 2.2. Cell Culture and Treatment

KGN cells were sourced from the NHC Key Laboratory of Study on Abnormal Gametes and Reproductive Tract (Anhui Medical University, Hefei, China). The cells were maintained in a culture medium that was complete, containing 10% fetal bovine serum and 1% solution of penicillin–streptomycin antibiotics. The cells were cultured at 37 °C in a humidified 5% CO_2_ atmosphere. The experiments were then conducted once cell confluence reached 60% to 80%. The KGN cells were exposed to varying concentrations of cisplatin (0, 20, and 40 μM) for 24 h, and the impacts of cisplatin on these cells were subsequently observed. For further experimentation, a concentration of 40 μM cisplatin was chosen. The cells were exposed to varying concentrations of UA (0, 1.0, 2.0, and 5.0 μM), CsA (0, 1.0, 5.0, and 10.0 μM), and Mel (0, 0.1, 1.0, and 10.0 μM) for 24 h, and then the assessment of cell viability was conducted using a CCK-8 assay. Based on the CCK-8 results, the optimal concentrations of CsA (1.0 μM), UA (5.0 μM), and Mel (1.0 μM) were determined. Subsequently, the KGN cells were divided into distinct treatment groups as follows: a blank control group, a Cis group (40 μM Cis), a Cis + CsA group (40 μM Cis + 1.0 μM CsA), a Cis+UA group (40 μM Cis + 5.0 μM UA), a Cis + Mel group (40 μM Cis + 1.0 μM Mel), and individual treatment groups for CsA (1.0 μM CsA), UA (5.0 μM UA), and Mel (1.0 μM Mel).

### 2.3. Apoptosis Assay

The Annexin Ⅴ-FITC/PI Apoptosis Detection Kit (BB-4101, BestBio, Shanghai, China) was utilized to detect the occurrence of apoptosis. The cellular density for plating KGN cells in 12-well dishes was set at 1 × 10^5^ cells per well. Upon achieving a cell confluence ranging from 60% to 80%, they were processed by the experimental procedure. The supernatant was collected, and subsequently, the cells were detached using trypsin without EDTA. Following this, the cells were subjected to centrifugation and were rinsed twice with cold phosphate-buffered saline (PBS). The cells were subsequently resuspended in 100 mL of 1× binding buffer. Afterward, they were stained with a combination of 5 μL of Annexin Ⅴ-FITC and 5 μL of PI. The staining process was carried out in the dark at room temperature for 15 min. Finally, the extent of apoptosis was determined via flow cytometry using the BD FACSVerse instrument (San Jose, CA, USA), with subsequent analyses conducted using FlowJo (Version 10) software.

### 2.4. Transmission Electron Microscopy (TEM) Analysis

The structure and formation process of mitochondrial autophagosomes and autophagic lysosomes were observed using TEM. The cells were first washed with PBS, digested with trypsin, and then collected using low-speed centrifugation. After discarding the supernatant, the cells were fixed with 1 mL of 2.5% glutaraldehyde (in 1× PBS) overnight at 4 °C in the dark. Following fixation, the samples were post-fixed in 2% osmium acid, washed, dehydrated using graded ethanol solutions, and finally embedded in epoxy resin. Sections measuring 45 nm in thickness were stained with both uranyl acetate and lead citrate. Subsequently, the ultrastructural characteristics of the samples were examined using a transmission electron microscope manufactured by JEOL (Tokyo, Japan).

### 2.5. Detection of Mitochondrial Membrane Potential and Intracellular ROS Content

The cellular density for plating KGN cells in 12-well dishes was set at 1 × 10^5^ cells per well. Upon achieving a cell confluence ranging from 60% to 80%, the cells underwent chemical treatment with varying concentrations. To evaluate the mitochondrial membrane potential (MMP), we employed the JC-1 Mitochondrial Membrane Potential Assay Kit (KTA4001, Abbkine, Taiwan, China). In detail, a 10 μL aliquot of the JC-1 working solution was introduced into each 1 mL of cell suspension (1 × 10^6^ cells), followed by gentle mixing. The mixture was allowed to incubate at 37 °C for 20 min in the dark. Afterward, the cells underwent two washes with pre-cooled PBS and were subsequently analyzed using flow cytometry (BD FACSVerse, USA).

For the detection of intracellular ROS content, DCFH-DA (CA1410, Solarbio, Beijing, China) was diluted in serum-free culture medium to a final concentration of 4 μM, with a dilution ratio of 1:4000. The cells were collected and then resuspended in the diluted DCFH-DA solution, achieving a cellular concentration ranging from 1 × 10^6^ to 10^7^ cells per mL. Subsequently, they were cultured in a 37 °C incubator designed for cellular growth, protected from light, for 20 min, with inversion every 3–5 min to ensure thorough probe–cell contact. Following incubation, the cells were rinsed twice with serum-free media to eliminate any residual extracellular DCFH-DA and were subsequently analyzed using flow cytometry (BD FACSVerse, USA). The resultant data were processed and interpreted utilizing FlowJo software.

### 2.6. Measurement of ATP Generation

The ATP assay kit (S0026, Beyotime, Shanghai, China) was utilized to detect the intracellular content of ATP. The cellular density for plating KGN cells in 12-well dishes was set at 1 × 10^5^ cells per well. Upon achieving a cell confluence ranging from 60% to 80%, the cells were subjected to chemical treatment utilizing distinct concentrations of the reagent. The cells were collected and then quantified. Cells were processed for lysis with 200 μL lysis buffer on ice, following the manufacturer’s guidelines. Afterward, the lysate was gathered and subjected to centrifugation at 4 °C for 5 min at a speed of 12,000× *g*. Subsequently, 20 μL of the resultant supernatant was aliquoted into a 96-well plate that had been preloaded with 100 μL of the ATP assay working solution. The absorbance was then measured using an enzyme marker. Based on the standard curve, the ATP concentration was determined as nmol of ATP per milligram of protein.

### 2.7. Western Blot Analysis

Following the manufacturer’s guidelines, the cells were lysed on ice using RIPA buffer supplemented with protease inhibitors. Subsequently, the resultant protein samples underwent fractionation using 12% SDS-polyacrylamide gel electrophoresis (SDS-PAGE) and were transferred onto PVDF membranes. Before incubation with the specific primary antibody at 4 °C overnight, these membranes were blocked with 5% non-fat milk for 1 h at room temperature. Afterward, the membrane was subjected to three consecutive washes with TBS containing Tween 20 (TBST), each lasting for 10 min. Subsequently, the membrane was incubated with a particular secondary antibody for 1 h at room temperature, followed by three 10-min washes with TBST. Visualization of the blot was achieved using an ECL ultrasensitive luminescent solution (BL523B, Biosharp, Hefei, China). As an internal control, either β-Actin or GAPDH was employed. The acquired results were analyzed utilizing image analysis software (ImageJ, Version 1.8.0, Bethesda, MA, USA).

### 2.8. Statistical Analysis

Each experiment was repeated at least three times, and the results were expressed as mean ± SD before statistical analysis using GraphPad Prism 9.5 software. The significance of differences was determined using one-way analysis of variance (ANOVA). Differences were considered statistically significant at *p* < 0.05.

## 3. Results

### 3.1. Cisplatin Inhibited Proliferation and Induced Apoptosis in KGN Cells

**Figure 1:**
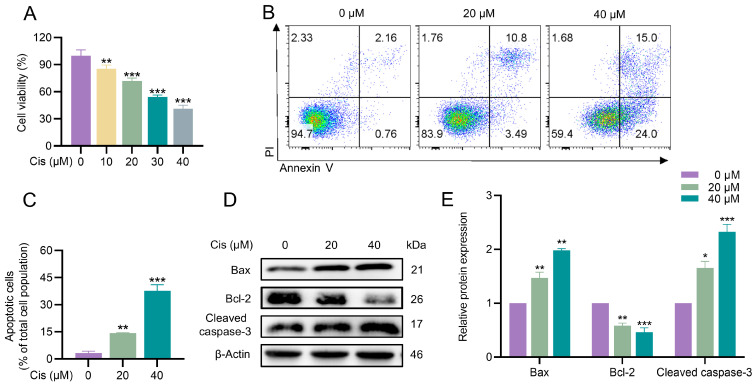
Cisplatin impaired cell viability and induced cell apoptosis in KGN cells. KGN cells were exposed to varying concentrations of cisplatin for 24 h. (**A**) The CCK-8 assay was utilized to ascertain relative cell viability. (**B**,**C**) Flow cytometry was employed to detect the apoptotic effects of cisplatin on KGN cells, with subsequent quantification of the apoptotic rates. (**D**,**E**) Western blot analysis was conducted to identify apoptosis-related proteins, including Bax, Bcl-2, and Cleaved caspase-3. The densitometric values of the bands were standardized with respect to β-Actin as an internal control. The results are presented as the mean ± standard deviation from at least three independent experimental repetitions. Significant differences from the control groups are denoted by an asterisk “*”, with statistical significance levels of * *p* < 0.05, ** *p* < 0.01, and *** *p* < 0.001.

To investigate the effects of cisplatin on KGN cell survival, we first assessed cell viability using the CCK-8 assay. KGN cells were treated with cisplatin at concentrations ranging from 0 to 40 μM (in 10 μM increments) for 24 h. The results revealed a dose-dependent reduction in cell viability compared to the control group (Figure 1A). Based on these findings, subsequent experiments were conducted using cisplatin concentrations of 0 μM, 20 μM, and 40 μM. Next, we examined the impact of cisplatin on apoptosis in KGN cells. Flow cytometry analysis demonstrated a significant increase in apoptosis in cisplatin-treated groups compared to the control group (Figure 1B,C). To further elucidate the underlying mechanisms, we analyzed the expression of apoptosis-related proteins using Western blotting. Cisplatin treatment led to a dose-dependent upregulation of pro-apoptotic proteins Bax and Cleaved caspase-3, whereas the expression of anti-apoptotic protein Bcl-2 was downregulated (Figure 1D,E). Collectively, these results indicated that cisplatin inhibited proliferation and induced apoptosis in KGN cells, potentially through the modulation of apoptosis-related protein expression.

### 3.2. Cisplatin Caused Mitochondrial Dysfunction in KGN Cells

**Figure 2:**
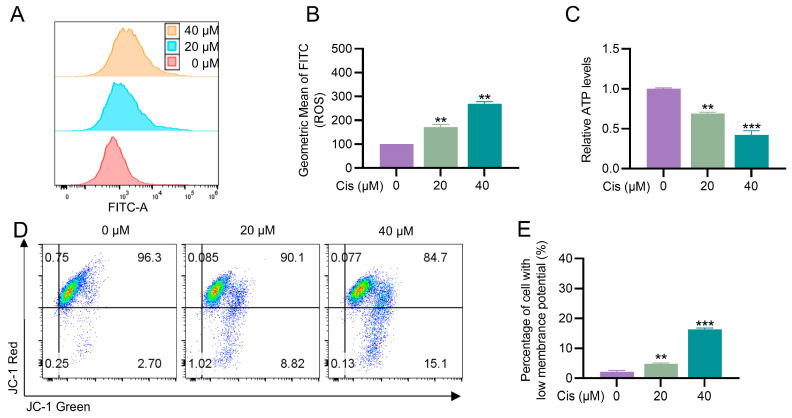
Cisplatin induces mitochondrial dysfunction in KGN cells. (**A**,**B**) Intracellular ROS levels were determined using flow cytometry. (**C**) ATP production for 24 h. (**D**,**E**) Detection of MMP using flow cytometry. “*” indicates a significant difference compared with the control group (** *p* < 0.01, and *** *p* < 0.001).

As central regulators of cellular metabolism and key determinants of cell fate, mitochondria play pivotal roles in maintaining cellular health, and their dysfunction has been implicated in various disease processes [24]. We therefore examined whether cisplatin affected mitochondrial function in KGN cells. We first measured intracellular ROS levels to assess oxidative stress. The results showed a dose-dependent increase in ROS production in cisplatin-treated cells compared to the control group (Figure 2A,B). Next, we evaluated the impact of cisplatin on mitochondrial function by assessing ATP production and mitochondrial membrane potential (MMP). Cisplatin treatment at 20 μM and 40 μM significantly reduced ATP levels by 31% and 57%, respectively (Figure 2C). Furthermore, cisplatin exposure led to a marked increase in the proportion of cells with depolarized mitochondria, as indicated by a reduction in MMP (Figure 2D,E). These findings demonstrate that cisplatin triggers oxidative stress and disrupts mitochondrial function in KGN cells, as evidenced by elevated ROS levels, reduced ATP production, and mitochondrial depolarization.

### 3.3. Cisplatin Induced Mitophagy in KGN Cells

**Figure 3:**
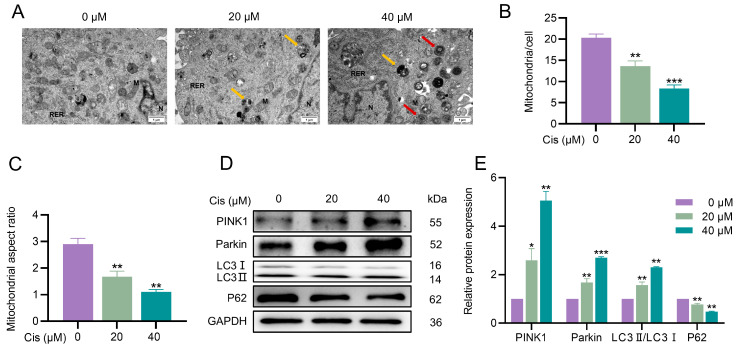
Cisplatin induced mitophagy in KGN cells. (**A**) Ultrastructure of mitochondria in KGN cells. Red arrows indicate mitochondrial autophagosomes. Yellow arrows indicate autophagic lysosomes. “M” indicates mitochondria, “N” indicates nucleus, and “RER” indicates rough endoplasmic reticulum. The scale bar is 1 μm. (**B**) Quantification of mitochondrial number. (**C**) Mitochondrial length/width ratio. (**D**,**E**) Protein levels of PINK1, Parkin, LC3I, LC3II, and P62 were detected using Western blot. GAPDH was used to normalize the densitometric values of the bands. “*” indicates a significant difference compared with the control group (* *p* < 0.05, ** *p* < 0.01, and *** *p* < 0.001).

Mitophagy is a selective autophagy process that removes damaged mitochondria, thereby maintaining mitochondrial homeostasis and preventing cellular dysfunction. Subsequently, we examined whether mitophagy was involved in contributing to cisplatin-induced cytotoxicity. We first examined the mitochondrial ultrastructure using transmission electron microscopy (TEM). Cisplatin treatment resulted in swollen mitochondria with disrupted cristae, accompanied by the presence of mitochondrial autophagosomes and autophagolysosomes, indicative of active mitophagy (Figure 3A–C). Given the central role of the PINK1–Parkin pathway in regulating mitophagy in mammalian cells, we next evaluated the expression of key mitophagy-related proteins. Cisplatin exposure significantly increased the LC3II/LC3I ratio, a marker of autophagosome formation, and upregulated the levels of PINK1 and Parkin proteins. In contrast, P62/SQSTM1, a selective autophagy substrate, showed significantly decreased expression, indicating enhanced mitophagic flux (Figure 3D,E). These findings demonstrated that cisplatin triggers mitophagy in KGN cells, as evidenced by ultrastructural changes in mitochondria and the activation of the PINK1–Parkin pathway.

### 3.4. Mitophagy Served as a Protective Mechanism in the Injury of KGN Cells Caused by Cisplatin

**Figure 4:**
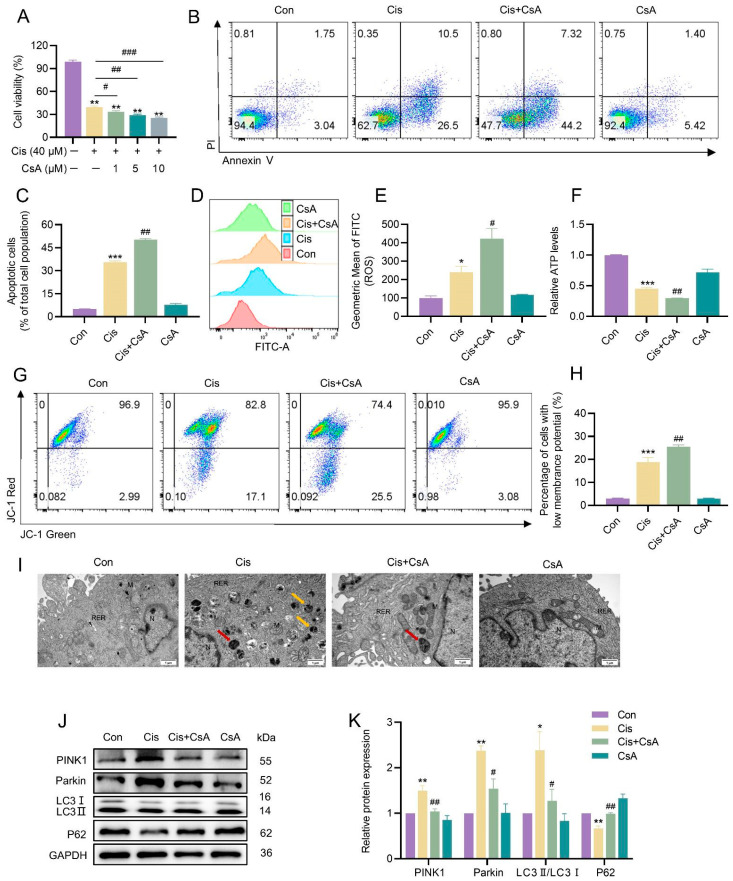
Inhibition of mitophagy by CsA aggravated cisplatin-induced mitochondrial damage and apoptosis in KGN cells. (**A**) The CCK-8 assay was employed to assess the impact of cisplatin and CsA on the viability of KGN cells, expressed as relative percentages. (**B**,**C**) Apoptosis was detected using flow cytometry in cells of different treatment groups, and the apoptosis rate was quantified. (**D**,**E**) Intracellular ROS levels were detected using flow cytometry. (**F**) The detection of ATP content. (**G**,**H**) The detection of MMP using flow cytometry. (**I**) TEM analysis of mitochondrial ultrastructure. The red arrows indicate mitochondrial autophagosomes. The yellow arrows indicate autophagic lysosomes. “M” indicates mitochondria, “N” indicates nucleus, and “RER” indicates rough endoplasmic reticulum. The scale bar is 1 μm. (**J**,**K**) Western blot analysis was utilized to detect the protein abundance of mitophagy-related factors, including PINK1, Parkin, LC3I, LC3II, and P62. The densitometric values of the bands were standardized concerning GAPDH as an internal control. The results are presented as the mean ± standard deviation from at least three independent experimental repetitions. Asterisks “*” denote statistically significant differences when compared to the controls, with thresholds set at * *p* < 0.05, ** *p* < 0.01, and *** *p* < 0.001. Meanwhile, a hash symbol “^#^” marks significant deviations from the cisplatin group, with ^#^ *p* < 0.05, ^##^ *p* < 0.01, and ^###^ *p* < 0.001 indicating the levels of significance.

Given the demonstrated activation of mitophagy by cisplatin in KGN cells, we investigated the specific role of mitophagy in cisplatin-induced apoptosis and mitochondrial damage. Mitophagy was inhibited by cyclosporine A (CsA), a mitophagy inhibitor. Compared to the cisplatin-only group, CsA treatment further reduced cell viability (Figure 4A) and increased apoptosis rates (Figure 4B,C). Additionally, CsA exacerbated mitochondrial dysfunction, as evidenced by elevated intracellular ROS levels (Figure 4D,E), further suppressed ATP production (Figure 4F), and accelerated the loss of MMP (Figure 4G,H). TEM analysis revealed a significant decrease in autophagosomes and autophagolysosomes in CsA-treated cells (Figure 4I). Consistent with these findings, CsA downregulated the expression of key mitophagy-related proteins, including LC3II/LC3I, Parkin, and PINK1, while upregulating P62 levels (Figure 4J,K).

**Figure 5:**
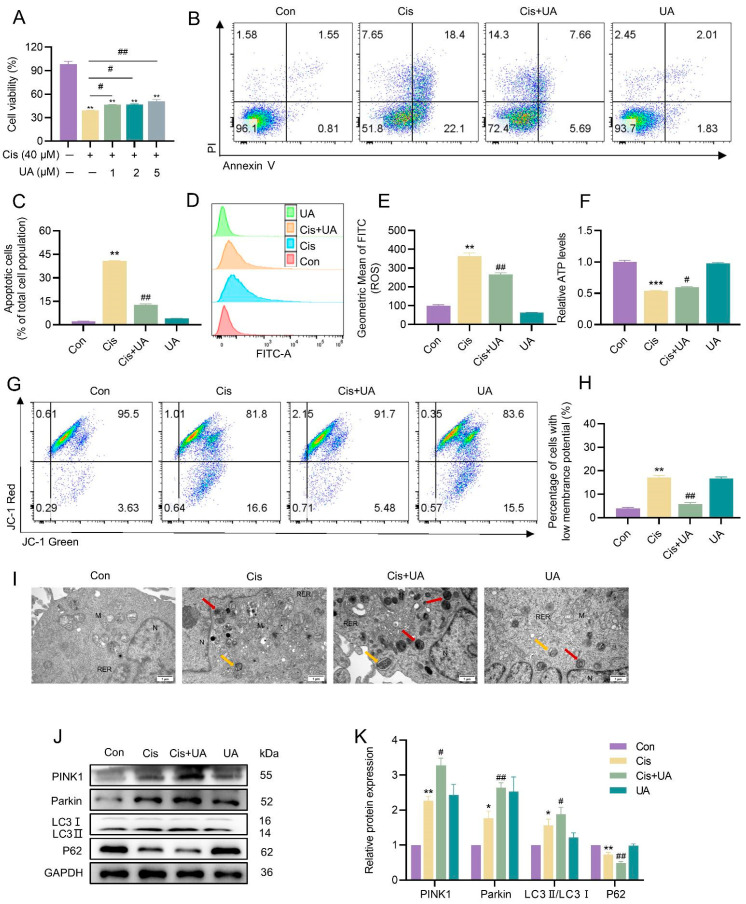
UA enhanced mitophagy and protected KGN cells from cisplatin-induced mitochondrial damage and apoptosis. (**A**) The CCK-8 assay was employed to assess the impact of cisplatin and UA on the viability of KGN cells, expressed as relative percentages. (**B**,**C**) Apoptosis was detected using flow cytometry in the cells of different treatment groups, and the apoptosis rate was quantified. (**D**,**E**) Intracellular ROS levels were detected using flow cytometry. (**F**) The detection of ATP content. (**G**,**H**) The detection of MMP using flow cytometry. (**I**) TEM analysis of the mitochondrial ultrastructure. The red arrows indicate mitochondrial autophagosomes. The yellow arrows indicate autophagic lysosomes. “M” indicates mitochondria, “N” indicates nucleus, and “RER” indicates rough endoplasmic reticulum. The scale bar is 1 μm. (**J**,**K**) Western blot analysis was utilized to detect the protein abundance of mitophagy-related factors, including PINK1, Parkin, LC3I, LC3II, and P62. The densitometric values of the bands were standardized concerning GAPDH as an internal control. The results are presented as the mean ± standard deviation from at least three independent experimental repetitions. Asterisks “*” denote statistically significant differences when compared to the controls, with thresholds set at * *p* < 0.05, ** *p* < 0.01, and *** *p* < 0.001. Meanwhile, a hash symbol “^#^” marks significant deviations from the cisplatin group, with ^#^ *p* < 0.05 and ^##^ *p* < 0.01 indicating the levels of significance.

In contrast, treatment with the mitophagy inducer urolithin A (UA) exerted opposing effects. Enhanced mitophagy significantly alleviated cisplatin-induced cytotoxicity, as demonstrated by increased cell viability (Figure 5A), reduced apoptosis (Figure 5B,C), and improved mitochondrial function (Figure 5D–H). TEM analysis confirmed a marked increase in autophagosomes and autophagolysosomes in UA-treated cells (Figure 5I). Furthermore, Western blotting revealed that UA upregulated the expression of PINK1, Parkin, and LC3II/LC3I proteins while downregulating P62 levels compared to the cisplatin-only group (Figure 5J,K). These results collectively demonstrated that mitophagy plays a protective role in mitigating cisplatin-induced mitochondrial damage and apoptosis in KGN cells.

### 3.5. Melatonin Activated Mitophagy and Attenuated Cisplatin-Induced KGN Cell Damage

**Figure 6:**
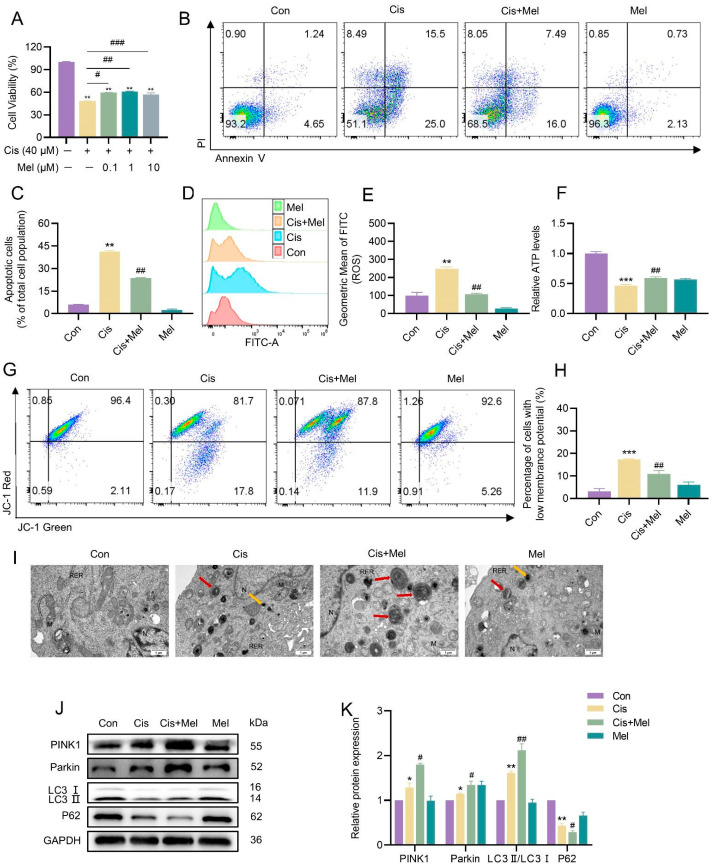
Melatonin attenuated cisplatin-induced mitochondrial damage and apoptosis in KGN cells by enhancing mitophagy. (**A**) The CCK-8 assay was employed to assess the impact of cisplatin and melatonin on the viability of KGN cells, expressed as relative percentages. (**B**,**C**) Apoptosis was detected using flow cytometry in cells of different treatment groups, and the apoptosis rate was quantified. (**D**,**E**) Intracellular ROS levels were detected using flow cytometry. (**F**) The detection of ATP content. (**G**,**H**) The detection of MMP using flow cytometry. (**I**) TEM analysis of the mitochondrial ultrastructure. The red arrows indicate mitochondrial autophagosomes. The yellow arrows indicate autophagic lysosomes. “M” indicates mitochondria, “N” indicates nucleus, and “RER” indicates rough endoplasmic reticulum. The scale bar is 1 μm. (**J**,**K**) Western blot analysis was utilized to detect the protein abundance of mitophagy-related factors, including PINK1, Parkin, LC3I, LC3II, and P62. The densitometric values of the bands were standardized concerning GAPDH as an internal control. The results are presented as the mean ± standard deviation from at least three independent experimental repetitions. Asterisks “*” denote statistically significant differences when compared to the controls, with thresholds set at * *p* < 0.05, ** *p* < 0.01, and *** *p* < 0.001. Meanwhile, a hash symbol “^#^” marks significant deviations from the cisplatin group, with ^#^ *p* < 0.05, ^##^ *p* < 0.01, and ^###^ *p* < 0.001 indicating the levels of significance.

The mitophagy inducer UA significantly alleviated cisplatin-induced mitochondrial damage and apoptosis; however, its clinical application remains limited. Previous studies have shown that melatonin, a clinically approved drug, can mitigate cisplatin-induced cytotoxicity in various disease models [25,26]. For instance, melatonin has been demonstrated to protect against cisplatin-induced ovarian damage and fertility decline in mice [27]. Despite these findings, the precise mechanism underlying melatonin’s protective effects remains unclear. Given emerging evidence that melatonin may promote mitophagy, we investigated whether melatonin’s cytoprotective effects against cisplatin-induced toxicity are mediated through activating mitophagy in KGN cells. To explore this, KGN cells were co-treated with cisplatin (40 μM) and melatonin (1.0 μM) for 24 h. Melatonin treatment significantly attenuated cisplatin-induced cellular damage, as evidenced by increased cell viability (Figure 6A) and reduced apoptosis (Figure 6B,C). Furthermore, melatonin decreased intracellular ROS levels (Figure 6D,E), enhanced ATP generation (Figure 6F), and restored MMP (Figure 6G,H), suggesting its role in improving mitochondrial function. To determine whether melatonin’s protective effects were associated with mitophagy activation, we assessed autophagosome and autolysosome formation. In addition, the expression levels of essential mitophagy-associated proteins were examined. Our results revealed an increase in autophagosome and autolysosome formation (Figure 6I). Additionally, protein levels of PINK1, Parkin, and the LC3II/LC3I ratio were upregulated, whereas P62 levels were downregulated (Figure 6J,K). These findings indicated that melatonin may protect against cisplatin-induced cytotoxicity by enhancing mitophagy and restoring mitochondrial homeostasis.

## 4. Discussion

Cisplatin is a cornerstone chemotherapeutic agent for treating malignant tumors; however, its reproductive toxicity at high doses poses a significant clinical challenge, particularly in young women who face chemotherapy-induced premature ovarian failure (POF) and subsequent fertility loss [28]. Although strategies to mitigate these adverse effects are urgently needed, the underlying mechanisms remain incompletely understood. Our study demonstrated that cisplatin triggered cell apoptosis and mitochondrial damage in KGN cells (a human ovarian granulosa cell line). Importantly, the targeted activation of mitophagy significantly attenuated these cytotoxic effects, suggesting mitophagy enhancement as a potential cytoprotective strategy against cisplatin-induced ovarian toxicity.

Cisplatin preferentially accumulates in mitochondria, where it induces mtDNA crosslinking and permeability transition pore opening, ultimately leading to bioenergetic collapse, oxidative stress, and mitochondrial dysfunction [29,30]. In this study, we demonstrated that cisplatin treatment significantly increased ROS production, caused MMP collapse, and reduced ATP production in KGN cells. These pathological changes likely lead to the accumulation of damaged mitochondria, which, if not efficiently removed through quality control mechanisms, may impair the functional integrity of the remaining mitochondrial population, thereby establishing a vicious cycle of mitochondrial dysfunction. Mitophagy is the selective autophagic clearance of damaged mitochondria and serves as a critical quality control mechanism to maintain mitochondrial homeostasis [31]. Here, we modulated mitophagy with pharmacological activators and inhibitors to investigate mitophagy’s role in cisplatin-induced KGN cell injury. UA, a mitophagy activator, not only enhanced PINK1–Parkin-dependent mitophagy but also restored mitochondrial function and reduced cisplatin-induced apoptosis in KGN cells. These findings align with previous studies showing UA’s therapeutic potential in conditions such as obesity-induced metabolic cardiomyopathy and intervertebral disk degeneration, where it activated mitophagy to alleviate damage [32,33]. Conversely, the mitophagy inhibitor CsA suppressed PINK1 and Parkin expression, reduced mitochondrial autophagosome formation, and exacerbated cisplatin-induced cell death. These results underscored the protective role of PINK1–Parkin-mediated mitophagy in countering cisplatin-induced damage, consistent with prior research demonstrating its cytoprotective effects in various pathological contexts [34].

The PINK1–Parkin pathway is a well-established mechanism for the selective removal of damaged mitochondria under physiological conditions in mammalian cells [35]. However, autophagy receptors, such as NIX, BNIP3, and FUNDC1, facilitate non-Parkin-mediated mitophagy pathways, which are crucial for maintaining mitochondrial quality [36]. For instance, BNIP3-mediated mitophagy has been shown to protect cardiomyocytes during myocardial ischemia/reperfusion injury [37]. Whether these non-Parkin-dependent pathways contribute to cisplatin-induced mitophagy in granulosa cells warrants further investigation.

The use of protective agents during chemotherapy is a promising strategy to minimize adverse effects. Melatonin, a potent antioxidant with anti-inflammatory and antitumor properties, has emerged as a potential adjunct therapy. Recent studies have not only revealed the protective effects of melatonin in a wide range of pathological conditions but have also specifically emphasized its potential application in chemoprotection. For example, melatonin can attenuate doxorubicin-induced myocardial toxicity, including oxidative stress, cellular pyroptosis, and apoptosis by activating the Sirt1/Nrf2 pathway [38]. Of particular note, melatonin was shown to attenuate cisplatin-induced ovarian damage and preserve fertility in a mouse model [27]. Importantly, various studies have highlighted the crucial roles of melatonin in regulating mitophagy [21]. For example, melatonin could enhance PINK1–Parkin-dependent mitophagy, thereby improving mitochondrial function and protecting against kidney injury in chronic kidney disease [23]. In our study, melatonin activated mitophagy, improved mitochondrial function, and protected KGN cells from cisplatin-induced damage. These findings provide a theoretical foundation for developing melatonin as a fertility-preserving agent during chemotherapy.

The molecular mechanisms by which melatonin affects mitophagy are intricate and context-dependent, involving multiple key signaling pathways. In addition to the classical PINK1–Parkin pathway, melatonin promotes the deacetylation process of mitochondrial proteins by activating deacetylases, such as SIRT3, which in turn enhances the activity of mitophagy-related proteins [39]. In addition, melatonin was able to utilize the SIRT1/FoxO1 signaling pathway to promote mitophagy [40]. However, several studies have demonstrated that melatonin can suppress mitophagy under specific physiological conditions [22,41]. The dichotomous regulation of melatonin on mitophagy may be related to the cellular redox status, the temporal and dose effects of melatonin, and the specificity of the cell type. Thus, these critical factors must be carefully considered when employing melatonin as a therapeutic agent for mitophagy-associated disorders.

Despite these insights, our study has limitations. First, we did not investigate the effects of cisplatin on ovarian tissue in vivo, leaving the in vivo mechanisms and therapeutic potential of mitophagy activation unexplored. Second, although melatonin protected granulosa cells from cisplatin-induced damage, its potential to confer resistance to cisplatin in tumor cells remains unclear. This represents a critical area for future research.

In conclusion, our study elucidated the protective role of mitophagy in cisplatin-induced damage to KGN cells and highlighted mitophagy activation as a novel therapeutic strategy for mitigating cisplatin-associated reproductive toxicity. These findings provide a foundation for future research and the development of targeted therapies to preserve fertility in patients undergoing chemotherapy.

## Data Availability

The data are available from the corresponding author upon reasonable request.
